# Single site-specific integration targeting coupled with embryonic stem cell differentiation provides a high-throughput alternative to in vivo enhancer analyses

**DOI:** 10.1242/bio.20136296

**Published:** 2013-10-07

**Authors:** Adam C. Wilkinson, Debbie K. Goode, Yi-Han Cheng, Diane E. Dickel, Sam Foster, Tim Sendall, Marloes R. Tijssen, Maria-Jose Sanchez, Len A. Pennacchio, Aileen M. Kirkpatrick, Berthold Göttgens

**Affiliations:** 1 Cambridge Institute for Medical Research and Wellcome Trust–MRC Cambridge Stem Cell Institute, University of Cambridge, Hills Road, Cambridge CB2 0XY, UK; 2 Genomics Division, Lawrence Berkeley National Laboratory, 1 Cyclotron Road, MS84-171, Berkeley, CA 94720, USA; 3 Centro Andaluz de Biología del Desarrollo (CABD), CSIC-Universidad Pablo de Olavide, Seville 41013, Spain

**Keywords:** ES cells, Enhancer, Haematopoiesis, Transcription

## Abstract

Comprehensive analysis of cis-regulatory elements is key to understanding the dynamic gene regulatory networks that control embryonic development. While transgenic animals represent the gold standard assay, their generation is costly, entails significant animal usage, and in utero development complicates time-course studies. As an alternative, embryonic stem (ES) cells can readily be differentiated in a process that correlates well with developing embryos. Here, we describe a highly effective platform for enhancer assays using an *Hsp68/Venus* reporter cassette that targets to the *Hprt* locus in mouse ES cells. This platform combines the flexibility of Gateway® cloning, live cell trackability of a fluorescent reporter, low background and the advantages of single copy insertion into a defined genomic locus. We demonstrate the successful recapitulation of tissue-specific enhancer activity for two cardiac and two haematopoietic enhancers. In addition, we used this assay to dissect the functionality of the highly conserved Ets/Ets/Gata motif in the *Scl+19* enhancer, which revealed that the Gata motif is not required for initiation of enhancer activity. We further confirmed that Gata2 is not required for endothelial activity of the *Scl+19* enhancer using *Gata2^−/−^ Scl+19* transgenic embryos. We have therefore established a valuable toolbox to study gene regulatory networks with broad applicability.

## Introduction

The intricate process of embryonic development involves dynamic interactions of transcription factors with gene regulatory elements within gene regulatory networks (GRNs) (Davidson, 2010; Pimanda and Göttgens, 2010). Deciphering the underlying mechanisms and identifying the participants of GRNs is therefore paramount for elucidating normal developmental processes. Key interactions involve the combinatorial binding of transcription factors to cis-regulatory elements, and fundamental insights can be acquired from their in silico and molecular dissection. Comprehensive experimental interrogation requires rigorous functional analyses for which the use of transgenic animals has historically been considered the gold standard assay.

For the mouse model this can be expensive in animal usage since variable copy-numbers of the transgene and integration positional effects require multiple transgenic lines in order to establish reproducible expression patterns. This variability can be circumvented by exploiting embryonic stem (ES) cell lines with a defective *hypoxanthine guanine phosphoribosyl transferase 1* (*Hprt*) gene, since strategies that restore *Hprt* function enable selection for single copy integration. As a ubiquitously expressed gene, *Hprt* resides in a favourable chromatin environment and it has been demonstrated that inclusion of tissue-specific promoter elements into *Hprt* targeting constructs results in transgene expression entirely under the control of exogenous regulatory elements. For example, *Flt-1*, *vWF* and *Tie-2* regulatory elements inserted as single-copy reporter transgenes into the *Hprt* gene locus all displayed appropriate expression patterns in transgenic mice (Evans et al., 2000; Minami et al., 2002). However, unlike the two-week time frame of analysing F0 transgenic embryos generated by microinjection, the generation of *Hprt* transgenic reporter mice takes a minimum of four months. Moreover, regardless of the procedure employed for acquiring transgenic mouse embryos their intra-uterine development complicates time-course studies.

Alternative in vitro methods are therefore highly desirable, not only to accelerate scientific progress, but also in light of significant animal welfare issues associated with large-scale generation of transgenic mouse lines. Mouse ES cells offer a distinct advantage due to the ease with which they can be manipulated, their ability to differentiate into cell types from all three germ layers, and for the way in which quantitative information of the activity of regulatory elements can be generated during in vitro differentiation time-courses. Using a *LacZ* reporter gene we have previously shown that the temporal activity of the well-characterised *Scl/Tal1* stem cell enhancer (*Scl+19*), inserted into the *Hprt* locus correlates well with endogenous gene expression (Smith, A. M. et al., 2008). However, while the traditional choice of *LacZ* reporter genes offers the advantage of performing histological studies with relative ease, the complex protocols required for flow cytometric analyses using this reporter limits the more advanced cellular experiments that are possible with alternative reporters such as GFP.

Here, we introduce a highly effective and adaptable toolkit that can be used to explore both wild type and perturbed enhancer activity at high-throughput, using mouse ES cell differentiation. We have replaced the *LacZ* reporter for a fluorescent reporter gene (*Venus-YFP*), enabling continuous time-course experiments, as well as substituting standard cloning strategies with Gateway® cloning (Hartley et al., 2000). This offers a rapid pipeline for more streamlined cloning together with a flexible and trackable reporter system. Here, we demonstrate the reproducibility of this approach for successful recapitulation of tissue-specific enhancer activity in both cardiac and haematopoietic lineages. Further advantages of using this technology become evident when we undertake focused dissection of Gata and Ets motifs that are known to influence the *Scl+*19 enhancer activity (Göttgens et al., 2002). We have gained new insights into the influence of individual transcription factors on tissue-specific activity.

## Results

### Reporter construction and minimal promoter selection

To streamline flow cytometric analyses of reporter gene activity, we replaced the *LacZ* reporter gene used previously (Smith, A. M. et al., 2008) with a yellow fluorescent reporter gene, *Venus* (Nagai et al., 2002) to generate an *Hprt* targeting cassette containing the *SV40* minimal promoter followed by the *Venus* reporter. However, a high proportion (∼80%) of differentiated ES cells targeted with the resulting *SV40/Venus* constructs showed YFP expression even without an enhancer (data not shown), indicating that the *SV40* minimal promoter is leaky in this context and therefore inappropriate for our assay. Recent large-scale transgenic studies of enhancers have made use of the *Hsp68* minimal promoter (Pennacchio et al., 2006; May et al., 2012; Visel et al., 2013), which has long been recognised as having a low background in enhancer assays (Kothary et al., 1989). We therefore replaced the *SV40* minimal promoter to produce an *Hsp68/Venus Hprt* targeting construct and then generated multiple independent *Hsp68/Venus* ES cell clones. In contrast to the *SV40/Venus* clones, *Hsp68/Venus* clones showed much lower background YFP (data not shown), thus suggesting that a suitable platform for enhancer analyses with fluorescent reporters had been established.

### Cardiac enhancers display activity in ES cell-derived beating cardiomyocytes

To test whether *Hsp68/Venus* constructs are effective for assessing tissue-specific enhancer activity in live cells, we selected previously characterised enhancers that drive reporter gene expression in the heart. We chose two enhancers using the VISTA Enhancer Browser (http://enhancer.lbl.gov; Visel et al., 2007); *mm75* and *mm77*. Enhancer *mm75* is located on chromosome 2 and is flanked by the bone morphogenetic protein BMP7 and *Tfap2d*, a transcription factor that is expressed in the developing heart (Zhao et al., 2003). Enhancer *mm77* is located on chromosome 12 between the transcription factor *Cux2* and the cardiac-specific gene *Myl2* (Chien et al., 1993). Both enhancers show strong and very specific activity in the developing heart (Visel et al., 2007).

**Fig. 1.**
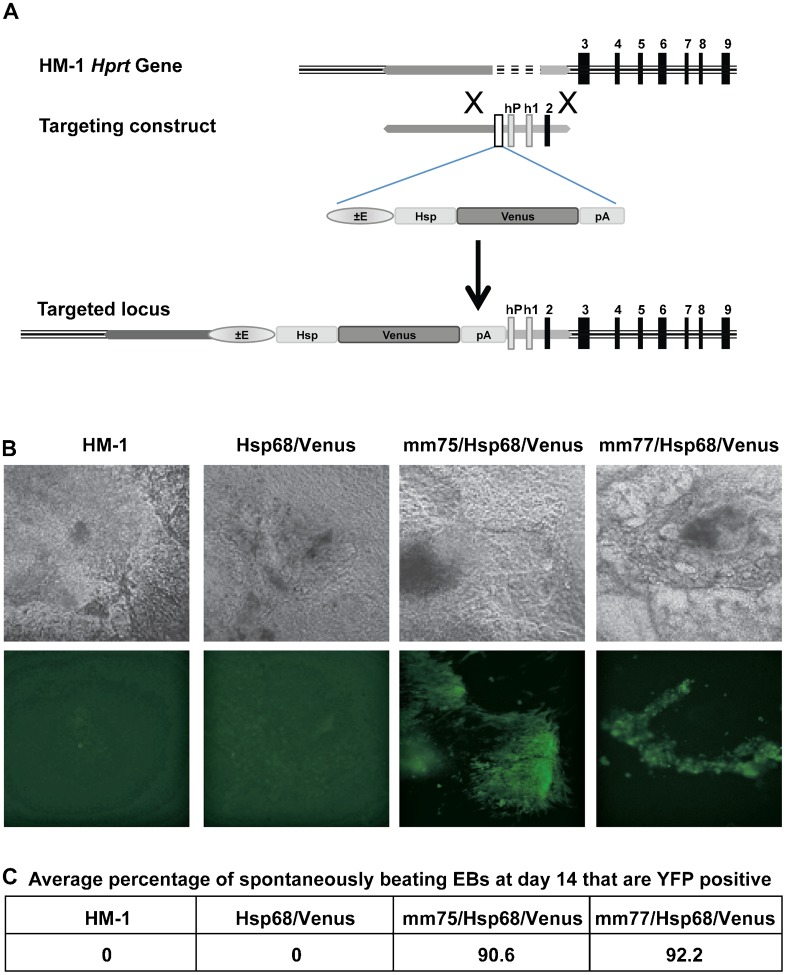
*Hprt* targeting strategy and cardiac enhancer activity in beating cardiomyocytes. (A) The *Hprt* locus in mouse HM-1 ES cells lacks the *Hprt* promoter, exon 1 and exon 2. The *Hprt* targeting vector contains the enhancer of interest, the *Hsp68* minimal promoter and *Venus* fluorescent reporter gene upstream of the human *HPRT* promoter, human exon 1 and mouse exon 2 and these are all flanked by *Hprt* locus homology arms. Homologous recombination of this targeting construct in HM-1 ES cells results in reconstitution of the *Hprt* locus and insertion of the *enhancer/Hsp68/Venus* reporter upstream of the promoter. Use of Hprt substrate analogues to select for HM-1 with deficient or reconstituted *Hprt* provides a stringent selection method for selection of correctly targeted clones. (B) Bright field (above) and fluorescent (below) images of representative beating embryoid bodies (EBs) at day 14, from HM-1, *Hsp68/Venus* control, *mm75/Hsp68/Venus* and *mm77/Hsp68/Venus* clones. (C) Average percentage of spontaneously beating EBs that are YFP positive at day 14 for the above clones, average of two independent differentiation experiments.

Using a cloning strategy that we have described previously (Smith, A. M. et al., 2008) we produced *mm75/Hsp68/Venus* and *mm77/Hsp68/Venus* reporter constructs and generated targeted HM-1 ES cell lines (Fig. 1A). After differentiating ES cells for eight days into embryoid bodies (EBs), cardiomyocytes are formed and spontaneously start beating. We therefore differentiated the ES cells for up to 14 days by which time cardiomyocytes are prevalent. We monitored YFP expression and found that, in agreement with expression patterns identified in the transgenic mice (Visel et al., 2007), both enhancers show highly specific activity in cardiomyocytes (Fig. 1B; supplementary material Movies 1–4). In order to quantitate this, we counted the number of spontaneously contracting EBs and then determined the percentage of these that expressed YFP. Over 90% of beating cardiomyocytes were YFP positive for each of the enhancer constructs (Fig. 1C). Importantly, no YFP fluorescence was observed in spontaneously contracting EBs derived from ES cell clones containing the enhancer-less *Hsp68/Venus* transgene. Robust cardiomyocyte expression of both heart enhancers therefore validated our in vitro enhancer assay as a potential alternative to conventional transgenic analyses.

### Increased efficiency with Gateway® cloning

Having established that the *Hsp68/Venus Hprt* targeting reporter gene constructs offer a powerful alternative to study tissue-specific enhancer activity, we next sought to improve the efficiency of construct generation. Gateway® Cloning Technology provides a highly efficient method of shuttling DNA sequences between multiple vector systems (Hartley et al., 2000) as it exploits recombination through specific attachment (att) sites identified from lambda bacteriophage interactions (Landy, 1989) and does not have the limitations of classic cloning strategies involving restriction sites. We therefore inserted *Hsp68/Venus* downstream of a Gateway® cassette and transferred this cassette into the *Hprt* targeting vector. Simple PCR amplification of enhancer sequences with primers that incorporate att sites now allows the generation of pDONR vectors from where the enhancers can be efficiently transferred into the new Gateway® adapted *Hsp68/Venus Hprt* targeting vector.

### A *Gfi1* enhancer shows activity within haematopoietic-fated mesoderm lineage

The transcription factor *Gfi1* is expressed in multiple tissues, including haematopoietic progenitors and stem cells, lymphoid and myeloid cells (Karsunky et al., 2002; Hock et al., 2004; Yücel et al., 2004; Rosenbauer and Tenen, 2007; Wilson et al., 2010a; Lancrin et al., 2012). We have previously identified an enhancer that is located around 35 kb upstream of *Gfi1* (referred to as the *Gfi1-35* enhancer) within an intron of the neighbouring gene *Evi5* (Wilson et al., 2010a). In transgenic mice this enhancer shows specific activity in the dorsal aorta and foetal liver, in a pattern that reflects endogenous gene expression (Wilson et al., 2010a; Lancrin et al., 2012). As the *Gfi1-35* enhancer is regulated by six haematopoietic transcription factors that are critical for haematopoiesis (Wilson et al., 2010b; Wilson et al., 2010a), this element is of particular interest in terms of gene regulatory networks that control the emergence of early blood progenitors. We therefore inserted the *Gfi1-35* element into the *Hsp/Venus* vector to further analyse its function using the ES cell in vitro differentiation model of haematopoiesis.

**Fig. 2.**
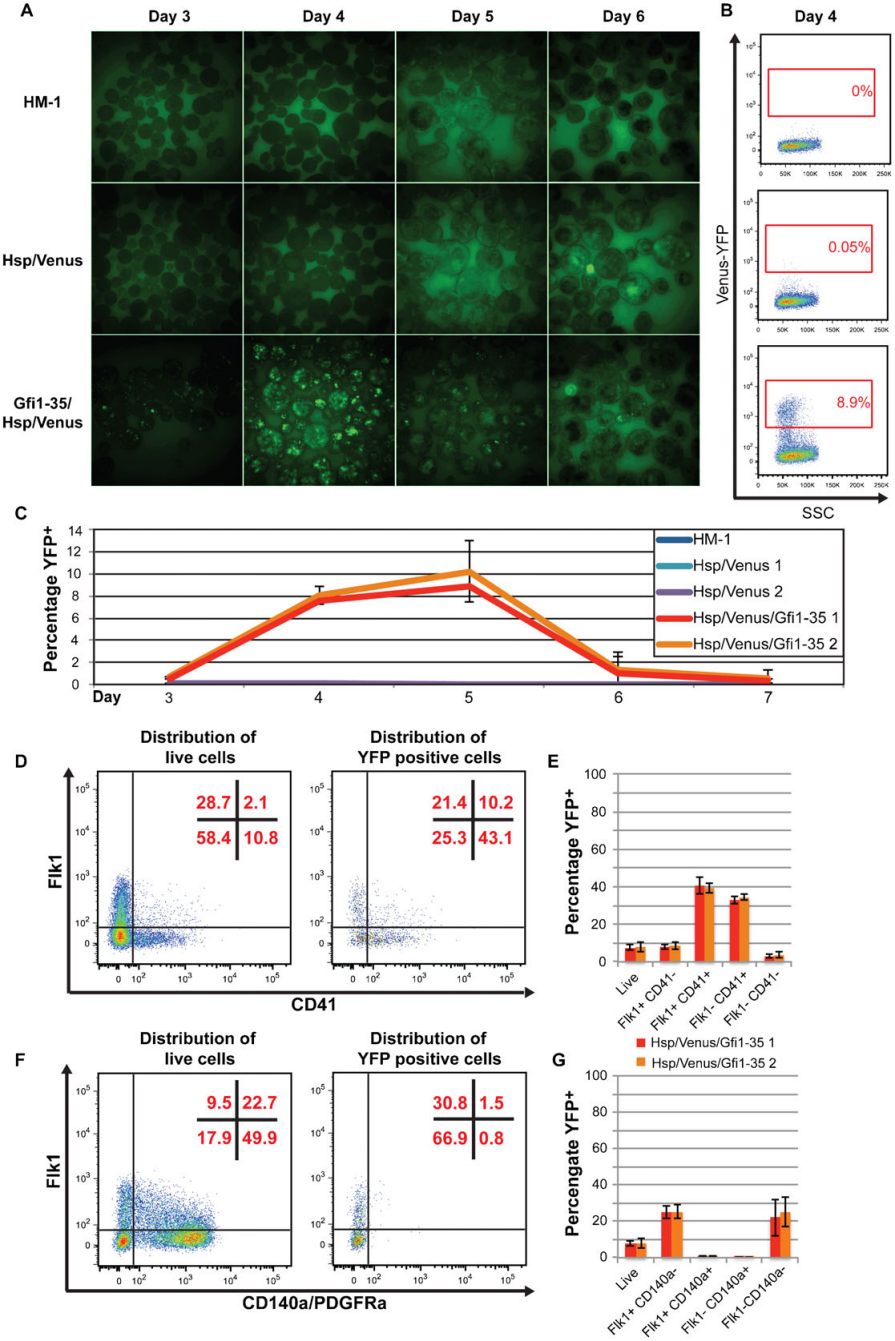
*Gfi1-35* activity marks haematopoietic-fated cells during embryoid body differentiation. (A) Fluorescent images of day 3–6 EBs from the representative clones of HM-1 (top), *Hsp/Venus* control (middle) and *Gfi1-35/Hsp/Venus* (bottom) lines. (B) Representative flow cytometry plots of Venus-YFP vs side scatter (SSC) for HM-1 (top), *Hsp/Venus* control (middle) and *Gfi1-35/Hsp/Venus* (bottom) day 4 EB cells with the YFP positive gate, and its percentage of the live cell population, shown in red. (C) Percentage of the YFP positive population (gating shown in B) from day 3–7 for HM-1 (blue), two representative *Hsp/Venus* control clones (aqua and purple) and two representative *Gfi1-35/Hsp/Venus* (red and orange) clones, showing average of three independent differentiation experiments ± standard deviation. (D) Representative flow cytometry plots showing distribution of *Gfi1-35/Hsp/Venus* day 4 EB cells in Flk1/CD41 quadrants for all live cells (left plot) and YFP positive cells only (right plot), with the percentage of cells in each quadrant shown in red. (E) Percentage of YFP positive cells in each of the Flk1/CD41 quadrants of (D) for *Gfi1-35/Hsp/Venus* (clone 1 in red, clone 2 in orange). Average of three independent differentiation experiments ± standard deviation. (F) As in (D), but for Flk1/CD140a quadrants. (G) As for (E), but for Flk1/CD140a quadrants in (F).

ES cells can readily differentiate towards the haematopoietic lineage in a process that closely resembles in vivo development (Keller et al., 1993), and which can be monitored using well-characterised cell surface markers (Mitjavila-Garcia et al., 2002; Mikkola et al., 2003). For example, the transition of Flk1 (*Kdr*) positive cells of the haemangioblast towards CD41 (*Itg2a*) positive haematopoietic progenitors from mouse embryonic stage E6.5–10.5, is reflected by the distribution of these cell surface markers during day 3–7 of ES cell differentiation (supplementary material Fig. S1). We initially followed YFP expression by fluorescent imaging of differentiating EBs from ES cells targeted with *Gfi1-35/Hsp/Venus* (and *Hsp/Venus* and HM-1 controls) from day 3 to 6 (Fig. 2A). YFP expression appeared brightest at day 4, but continued to day 6.

To quantify this YFP expression, we disaggregated EBs and analysed YFP expression by flow cytometry, initially at day 4. A distinct YFP positive population was apparent above background levels in the controls (Fig. 2B). We determined the size of the YFP^+^ population by flow cytometry from day 3 to 7, and found that it peaks at day 5 at 8–10%. However, as seen by fluorescent microscopy, YFP expression level was highest at day 4 (Fig. 2C). At day 4, these YFP^+^ EB cells are found within Flk1^+^CD41^−^, Flk^+^CD41^+^ and Flk1^−^CD41^+^ populations (specifically enriched in the later two populations), but not the CD140a^+^ cardiac lineage population (Fig. 2D), consistent with the specific activity of the *Gfi1-35* enhancer in haematopoietic specification, and the previously reported embryonic staining pattern in mouse embryos (Wilson et al., 2010a; Lancrin et al., 2012). However, previous analyses of the *Gfi1-35* enhancer were limited to a single time point (E11.5 of mouse embryo development), and analyses of endogenous *Gfi1* expression using knockin mice is hampered by the limited numbers of early blood progenitors that can be obtained from early mouse embryos. By contrast, as well as readily permitting the generation of early blood progenitor populations, the ES cell-based *Gfi1-35* reporter system enables us to follow the entire developmental program from embryonic mesoderm to multi-potential blood progenitors. As a consequence, our new *Gfi1-35* enhancer reporter system provides an unprecedented level of detail about enhancer activity during haematopoietic specification, and suggests that *Gfi1-35* is active in Flk1^+^ mesoderm fated to haematopoietic rather than cardiac specification, prior to CD41 expression, remaining active during the specification of committed early haematopoietic cells (Flk1^−^CD41^+^).

### The *Scl+19* enhancer marks mesoderm with haematopoietic and cardiac potential

To further assess the ability of the *Hsp68/Venus Hprt* targeting approach to recapitulate in vivo enhancer data and dissect the functionality of regulatory elements, we chose to concentrate on the *Scl+19* enhancer, due to its simple and highly conserved Ets/Ets/Gata motif, well-characterised activity in transgenic embryos and luciferase assays (Göttgens et al., 2002; Göttgens et al., 2004; Silberstein et al., 2005; Spensberger et al., 2012). *Scl/Tal1* is a critical transcription factor for developmental patterning in the mouse conceptus, necessary for primitive and definitive haematopoiesis (Shivdasani et al., 1995; Porcher et al., 1996; Robb et al., 1996; Barton et al., 2001; Kassouf et al., 2008) and antagonising cardiac fate of embryonic endothelium (Ismailoglu et al., 2008; Van Handel et al., 2012). Its correct spatiotemporal expression is therefore critical for normal development. In transgenic mouse embryos the *Scl+19* enhancer is active in embryonic endothelium, haemangioblasts, and committed haematopoietic progenitors (Sánchez et al., 1999; Göttgens et al., 2002; Silberstein et al., 2005).

**Fig. 3.**
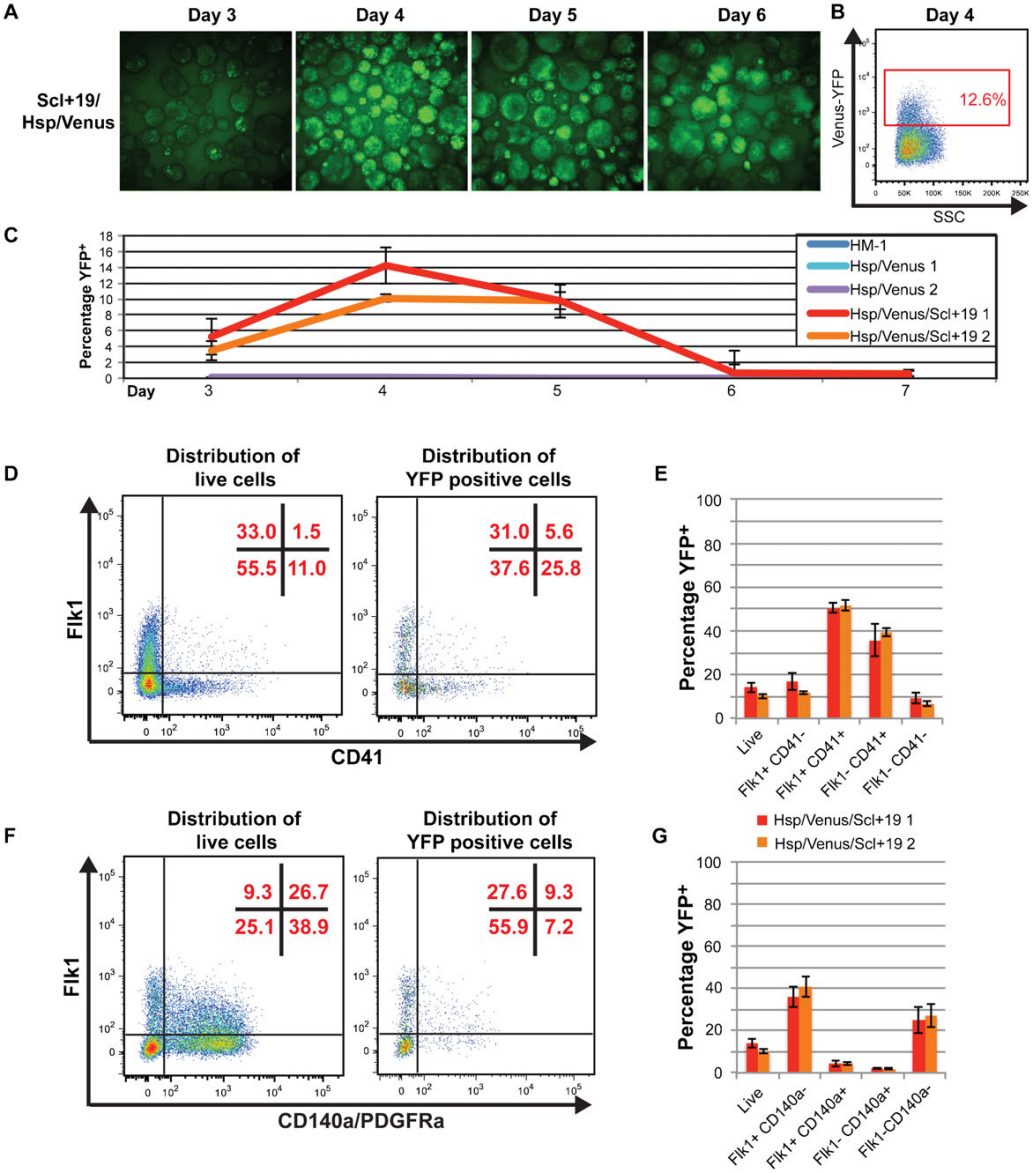
*Scl+19* activity marks haematopoietic cells and cardiac mesoderm in differentiating embryoid bodies. (A) Fluorescent images of day 3–6 EBs from a representative *Scl+19/Hsp/Venus* clone. (B) Representative flow cytometry plots of Venus-YFP vs side scatter (SSC) of day 4 EBs for *Scl+19/Hsp/Venus* line with the YFP positive gate, and its percentage of the live cell population, shown in red. (C) Percentage of the YFP^+^ population (gating shown in B) from day 3–7 for HM-1 (blue), two representative *Hsp/Venus* control clones (aqua and purple) and two representative *Scl+19/Hsp/Venus* clones (red and orange), showing the average of three independent differentiation experiments ± standard deviation. (D) Representative flow cytometry plots showing distribution of *Scl+19/Hsp/Venus* day 4 EB cells in Flk1/CD41 quadrants for all live cells (left plot) and YFP positive cells only (right plot), with the percentage of cells in each quadrant shown in red. (E) Percentage of YFP positive cells in each of the Flk1/CD41 quadrants of (D) for *Scl+19/Hsp/Venus* (clone 1 in red, clone 2 in orange). Average of three independent differentiation experiments ± standard deviation. (F) As in (D), but for Flk1/CD140a quadrants. (G) As for (E), but for Flk1/CD140a quadrants in (F).

Using the Gateway® strategy outlined above we targeted the *Scl+19* enhancer into the *Hprt* gene locus in HM-1 ES cells. Analysis of differentiating EBs using fluorescent imaging suggested the *Scl+19* enhancer was active from day 3 to 6 with a large percentage of EB cells expressing YFP during this period (Fig. 3A). To further investigate the *Scl+19* activity, we disaggregated the EBs at day 4 and analysed YFP expression by flow cytometry. As with *SV/LacZ* reporters of the *Scl+19* in transgenic animals, baseline fluorescence of enhancer-targeted cells was above the HM-1 control (Fig. 2B, Fig. 3B). However, YFP^+^ cells were readily identifiable as a distinct population (Fig. 3B). We further analysed this YFP^+^ population during the EB differentiation by flow cytometry. Similar to the *Gfi1-35*, the YFP^+^ population was seen from day 3 to 5, peaking at day 4 when 10–14% of the differentiating cells were YFP^+^ (Fig. 3C). By day 6, only background level YFP expression was seen.

We next looked at the distribution of YFP^+^ cells at day 4 EB, using the surface markers Flk1, CD140a and CD41 to chart developmental progression towards a haematopoietic fate. YFP^+^ cells were enriched in the Flk1^+^CD41^−^, Flk1^+^CD41^+^ and Flk1^−^CD41^+^ populations during haematopoietic specification. YFP^+^ cells were relatively depleted amongst CD140a^+^ cells (65.6% of live cells were CD140a^+^ compared with 16.5% of YFP^+^ cells (Fig. 3E)). While this result suggests some enrichment for activity in the non-cardiogenic mesoderm this was much less pronounced than for the *Gfi1-35* enhancer, where only 2.3% of YFP^+^ cells expressed CD140a (Fig. 2E). Importantly, consistent differences between the *Scl+19* and *Gfi1-35* enhancers were observed using multiple independent ES cell clones, with the *Scl+19* enhancer showing relatively high activity in developmentally more immature cells.

The cellular resolution afforded by the *Hsp68/Venus* reporter system therefore provides detailed developmental stage-specific information on enhancer activity that would be very difficult to obtain using transgenic mice.

### Quantitative dissection of the *Scl+19* enhancer by motif mutations

Having demonstrated the ability to identify distinct activity patterns for two different haematopoietic enhancers, we next explored the utility of the *Hsp68/Venus Hprt* targeting approach to compare activities of wild type and mutant enhancers. Transcription factor binding events at regulatory elements are predicted by the presence of conserved binding motifs and the mutation of such motifs allows the specific activity of a class of transcription factors to be determined. However, current methods of mutational analysis are limited to population level luciferase assays in transformed cell lines or qualitative analysis in transgenic embryos. Previous analysis of the *Scl+19* by these methods suggested that mutation of any of the two highly conserved Ets motifs or the single Gata motif results in loss of activity (Göttgens et al., 2002). Here we exploit the aforementioned advantages of the *Hprt* targeting approach and use this for quantitative analyses to compare the effects of motif mutations on enhancer activity. The YFP reporter facilitates analyses at the single cell level and the close resemblance of differentiating cell types in vitro and in vivo enable us to model the effects in the developing embryo.

**Fig. 4.**
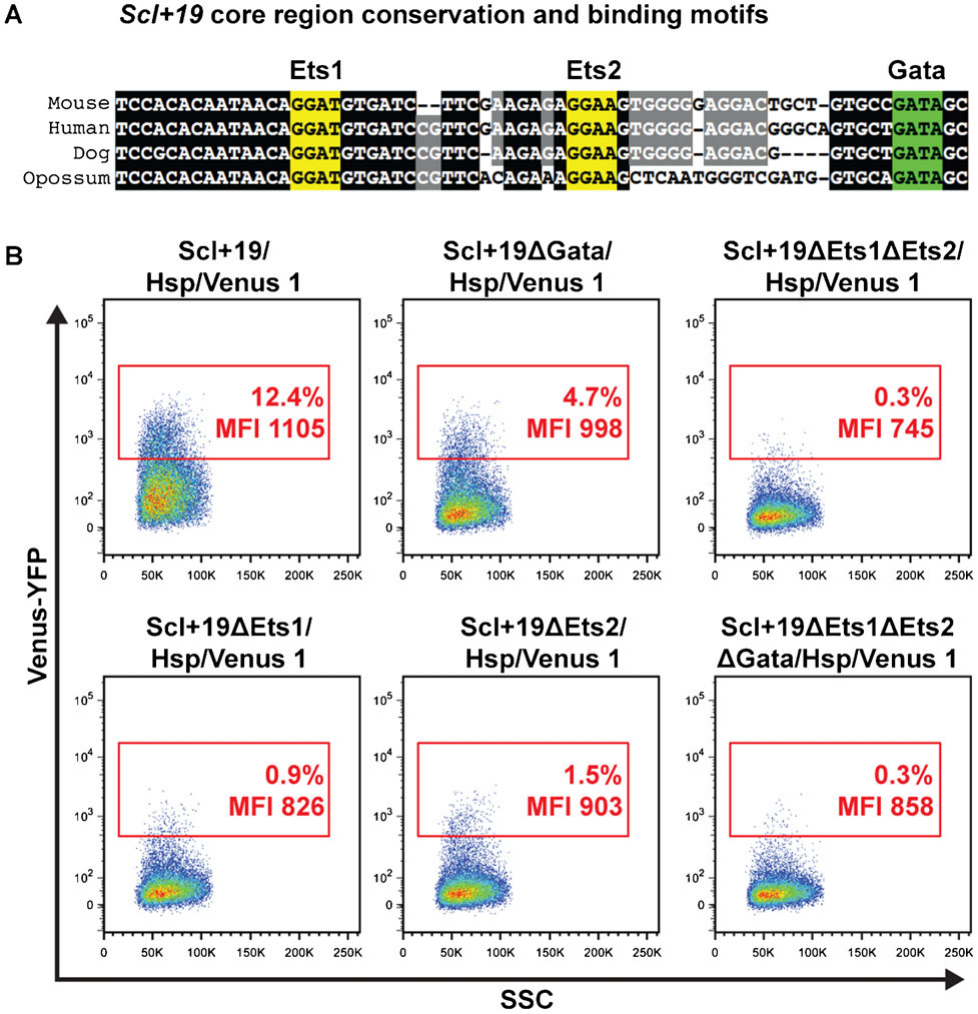
Dissection of the *Scl+19* enhancer by motif mutation. (A) Sequence conservation of the *Scl+19* core region in mouse, human, dog and opossum, with Ets motifs highlighted in yellow and the Gata motif highlighted in green. (B) Representative flow cytometry plots of Venus-YFP vs side scatter (SSC) of day 4 EBs for *Scl+19/Hsp/Venus* and *Scl+19* mutant clones with the YFP positive gate, its percentage of the live cell population and mean fluorescence intensity (MFI), shown in red.

To assess the contribution of the Ets and Gata motifs found within the *Scl+19* (Fig. 4A), we introduced *Scl+19* motif mutations created previously (Göttgens et al., 2002) into *Hsp/Venus Hprt* targeting constructs using Gateway® cloning, and targeted these mutated enhancers to the *Hprt* locus in HM-1 ES cells. As wild type *Scl+19* activity peaks at day 4 of the differentiation, we assessed the effects of the motif mutations on YFP expression at this time point by flow cytometry (Fig. 4B). Mutation of the Gata motif (*Scl+19ΔGata*/*Hsp/Venus*) reduced the percentage of YFP^+^ cells from ∼12% to ∼5%. Mutation of the Ets motifs (*Scl+19ΔEts1/Hsp/Venus* and *Scl+19ΔEts2/Hsp/Venus*) individually caused a more severe reduction in the YFP^+^ population to 0.9 and 1.5%, respectively. Mutation of all three motifs (*Scl+19ΔEts1ΔEts2ΔGata/Hsp/Venus*) or only both Ets motifs simultaneously (*Scl+19ΔEts1ΔEts2/Hsp/Venus*) caused complete loss of the YFP signal to background levels. In accordance with the reduction in the size of the YFP^+^ populations of the *Scl+19* mutants, the mean fluorescent intensity (MFI) is also least affected in the *Scl+19ΔGata*/*Hsp/Venus* (998 vs 1105 in the wild type *Scl+19/Hsp/Venus*), while mutation of the Ets motifs results in an MFI that is more similar to the *Scl+19ΔEts1ΔEts2ΔGata/Hsp/Venus* (745–903 vs 858). To determine whether loss of the Gata motif was cell-type specific, we analysed YFP expression within CD41/Flk1 quadrants at day 4 EB (supplementary material Fig. S2). Relative to wild-type *Scl+19* activity, YFP expression was lost in approximately half of Flk1^+^CD41^−^ and Flk1^+^CD41^+^ populations, and approximately three-quarters of Flk1^−^CD41^+^ cells. This trend suggests the Gata motif may be more important, although still not necessary, for *Scl+19* activity in committed CD41^+^ haematopoietic cells.

The use of single copy Venus-YFP reporter integration by this assay therefore affords a quantitative dissection of motif contribution to enhancer activity. Combined, these data suggest that all three motifs within the *Scl+19* are necessary for its full activity. However, the motifs are dispensable for partial *Scl+19* activity, as YFP^+^ cells are seen in all single motif mutation lines, but at a lower percentage. This observation suggests that increased transcription factor occupancy at the *Scl+19* enhancer increases the likelihood that the enhancer will become active in a given cell. Moreover, mutation of the Gata motif caused a relatively mild reduction compared to mutation of the Ets motifs, suggesting that Gata factors are not critically required to activate the *Scl+19*.

### Gata2 is not required for endothelial activity of the *Scl+19* enhancer in vivo

**Fig. 5.**
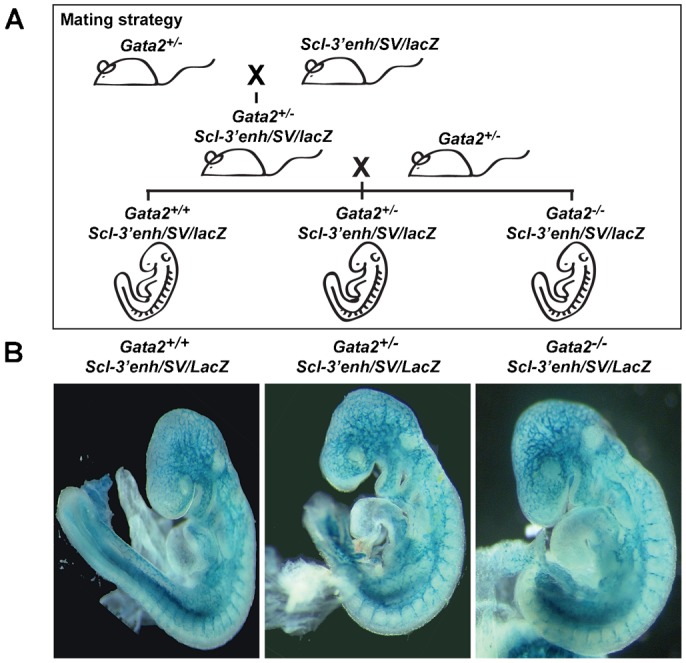
*Gata2* is not required for endothelial activity of the *Scl+19* enhancer in E9.5 transgenic mouse embryos. (A) *Gata2^+/−^* mice were crossed with mice carrying an *Scl+19* reporter (*Scl-3′enh/SV/lacZ*) to produce *Gata2*^+/−^ *Scl-3′enh/SV/lacZ* mice. These were re-crossed with *Gata2^+/−^* mice, from which E9.5 embryos were collected and genotyped for *Gata2* and *Scl-3′enh/SV/lacZ* phenotypes. (B) Representative whole mount images of E9.5 mouse embryos, genotypes shown above, stained for lacZ expression using Xgal.

The observation that initiation of *Scl+19* activity during ES cell differentiation did not require an intact Gata motif was surprising given that we had shown previously that *Gata2* binding is important for *Scl+19* enhancer activity in haematopoietic cells (Göttgens et al., 2002; Pimanda et al., 2007). *Gata2* was originally identified as an endothelial transcription factor (Wilson et al., 1990), and subsequently shown to be expressed in haemangioblasts and blood stem/progenitor cells (Suzuki et al., 2006; Lugus et al., 2007). To investigate whether *Gata2* is dispensable for induction of the *Scl+19* enhancer in vivo, we crossed transgenic mice carrying an *Scl+19* enhancer *LacZ* reporter (*Scl-3′enh/SV/lacZ*) (Sánchez et al., 1999; Sánchez et al., 2001) with *Gata2*^+/−^ mice, and then generated *Gata2*^+/+^, *Gata2*^+/−^ or *Gata2*^−/−^ E9.5 transgenic mouse embryos carrying the *Scl+19* reporter (Fig. 5A).

*LacZ* staining showed *Scl+19* activity within the endothelium was similar in *Gata2*^+/−^ and *Gata2*^−/−^ E9.5 embryos compared to wild-type embryo staining, thus demonstrating *Gata2* is not required for *Scl+19* activity at this early stage of development. Due to the embryonic lethality of *Gata2* deletion, the role of *Gata2* in the activity of the *Scl+19* at later stages of development, such as during definitive haematopoiesis, could not be determined. Nevertheless, in vivo analysis at E9.5 is consistent with our analyses of *Hsp68/Venus* reporter ES cell lines, which had shown Ets activity to be more important than Gata factors to activate the *Scl+19* enhancer.

## Discussion

Here, we have validated a novel ES cell-based enhancer assay that combines an efficient Gateway® cloning and *Hprt* gene targeting strategy with the flexibility and speed of analysis afforded by in vitro ES cell differentiation models and live cell analysis capability via YFP reporter tracking. This robust system additionally offers quantitative analysis of enhancer motif mutations to dissect regulatory inputs. Beyond these technological advantages, this assay also provides significant financial and animal welfare benefits over current costly transgenic mouse methodologies.

While we present here only the use of our new *Hprt* targeting constructs for analysis of enhancers in cardiac and haematopoietic lineages, it is not limited to these. Numerous other cell types can be generated in EBs by altering the culture conditions, including endothelium, adipocytes, skeletal and smooth muscle, neurons, and hepatocytes, in differentiation pathways that are thought to mimic normal development (reviewed elsewhere (Höpfl et al., 2004) and summarised in supplementary material Table S1). We therefore believe this system has broad applicability to analyses and dissection of enhancer activity in many developmental pathways and provides an attractive alternative to current transgenic mouse based methods.

While surface marker expression during embryonic development of the haematopoietic system has been well defined, this is not the case for other cell types. However, numerous tissue-specific cis-regulatory elements have been identified during embryogenesis by the VISTA Enhancer Browser project (Visel et al., 2007). Combination of this knowledge with our *Hsp68/Venus* reporter ES cell targeting strategy allows YFP expression to be used as a surrogate marker for lineage commitment, providing a robust read out for optimisation of ES cell differentiation protocols or screening surface marker expression during organogenesis. The approach described here could also be applied to human development where it could find wide application in regenerative medical research, by adaption of the targeting approach. The *HPRT* (Zwaka and Thomson, 2003), *ROSA26* (Irion et al., 2007) and *OCT4* (Hockemeyer et al., 2009) loci have all been successfully targeted in human ES cells. More recently, the AAV1 site within protein phosphatase 1 regulatory subunit 12C (PPP1R12C) (Samulski et al., 1991) has been shown to be the preferred site for gene targeting in human ES and iPS cells (Smith, J. R. et al., 2008; Hockemeyer et al., 2009). PPP1R12C is ubiquitously expressed in most cell types and the gene locus is in an open and active chromatin state permitting high levels of transgene expression. Moreover, chromatin insulators at the AAVS1 site ensure that transgene insertion does not perturb neighbouring gene expression and that the targeted transgene expression is tightly controlled (Lombardo et al., 2011).

Using our novel enhancer assay, we present evidence that both the *Scl+19* and *Gfi1-35* are active from the Flk1^+^ mesoderm stage through to committed CD41^+^ haematopoietic cell stages of haematopoietic specification. *Gfi1-35* and *Scl+19* enhancer activities show similar kinetics and specificities, although the *Scl+19* is active earlier in the differentiation, with YFP^+^ cells seen from day 3 of the EB differentiation while *Gfi1-35* activity is only seen from day 4 of the EB differentiation. This later activity of the *Gfi1-35* is not unsurprising considering *Scl* is thought to be an important upstream regulator of this element (Wilson et al., 2010b). While *Scl* plays a critical early role in haematopoiesis, where it is necessary for haemangioblast commitment from Flk1^+^ mesoderm, *Gfi1* is thought to play a later role in haematopoiesis, during Flk1^+^CD41^+^ to committed CD41^+^ haematopoietic cells (Lancrin et al., 2012). However, our data suggest the *Gfi1-35* is active as early as the Flk1^+^CD41^−^ stage, implying the haematopoietic transcription factor network that includes *Gfi1-35* is active prior to CD41 expression during haematopoietic specification, and may indeed mark cells fated to this lineage.

Unlike the broad haematopoietic and endothelial staining patterning of the *Scl+19* seen in Fig. 5B and (Göttgens et al., 2002), *Gfi1-35* activity marks only haematopoietic clusters in the dorsal aorta and cells in the foetal liver of developing embryos (Lancrin et al., 2012). Consistent with this, our data show greater specificity of *Gfi1-35* activity compared to the *Scl+19* in day 4 EBs for cells undergoing commitment to the haematopoietic lineage. Furthermore, *Scl+19* activity is seen in 16% of CD140a^+^ cells at this time point, when CD140a expression is thought to mark prospective cardiac mesoderm and progenitors. Interestingly, *Scl* is thought to play a role in patterning of cardiac mesoderm (Van Handel et al., 2012), with *Scl*^−/−^ endocardium spontaneously beating while *Scl* overexpression inhibits cardiac differentiation in EBs (Ismailoglu et al., 2008). Our data suggest *Scl+19* is active in a subset of cardiac mesoderm, where *Scl* expression likely plays a role in cardiac vs endothelial fate.

In summary, we have developed an efficient and robust enhancer assay as an alternative to current transgenic mouse methods and have validated its ability to recapitulate tissue-specific enhancer activity in both cardiac and haematopoietic lineages.

## Materials and Methods

### Targeting vector construction

To generate a reporter gene construct containing *Venus* downstream of an *Hsp68* promoter, the *SV40* promoter was cut out of *SV40/Venus/pA KS* using a HindIII restriction digest, end filling then digesting with NotI. We then cloned in the *Hsp68* promoter, which was isolated from *Hsp68LacZ KS*, using a BamHI digest, followed by end filling and digesting with NotI. Subsequently this *Hsp68/Venus/PA* construct was digested with SphI, end filled and digested with NotI in preparation for cloning the cardiac enhancers *mm75* and *mm77*, both of which were available as *HspLacZ KS* reporter gene constructs (Lawrence Berkeley National Laboratory (Visel et al., 2007)). These constructs were digested with BamHI (mm75) or SmaI (mm77), end filled and then digested with NotI before cloning into the linearised *Hsp68/Venus/PA* vector. The resulting enhancer *Hsp68/Venus* reporter cassette was then cloned as a NotI-Asp718 (blunt ended) fragment into the *Hprt* targeting construct *pMP8NEBdeltalacZ* (Misra et al., 2001) digested with MluI (end filled) and NotI.

To adapt the cloning to Gateway technology, the reporter cassette was isolated from the *Hsp68/Venus/PA* construct as a SphI blunt ended fragment and cloned immediately downstream of the Gateway® Cassette A (Invitrogen Life Technologies). The *Hsp68/Venus/Gateway® Cassette* was then cut out as a XhoI fragment, filled and blunt end cloned into the *Hprt* targeting construct pSKB1 (Bronson et al., 1996), linearised with MluI and filled in. The resulting targeting (destination) vector, *pSKB1-GW-Hsp68-Venus* (*Hsp/Venus* Gateway®) contained the Gateway® reporter gene cassette flanked by longer *Hprt* homology arms than the above (*pMP8NEBdeltalacZ*) targeting construct thus enabling greater recombination efficiency.

The *Gfi-35* element was PCR amplified using primers with attB sequences (underlined) upstream of enhancer specific sequence (gfi1_35attb1F GGGGACAAGTTTGTACAAAAAAGCAGGCTGAGGTTTTTAAGGCAGTGAATCAT, gfi1_35attb1R GGGGACCACTTTGTACAAGAAAGCTGGGTCACTAGAACCGAGTGCTGGA). This was then gel purified (Qiagen 78704) and used for BP recombination into the pDONR^TM^221 vector (Invitrogen Life Technologies). Successfully generated Gfi-35 pDONR^TM^221 vectors were recombined with the *Hsp/Venus* Gateway® destination vector using LR clonase (Invitrogen Life Technologies).

The *Scl+19* wild type and mutated sequences were cloned into the *Hsp/Venus* Gateway® targeting construct from *SV/Luc* constructs from (Göttgens et al., 2002) primers with attB sequences (underlined), GGGGACAAGTTTGTACAAAAAAGCAGGCTATATTAATCCCTCACTCAACAGCA and GGGGACCACTTTGTACAAGAAAGCTGGGTTGAGGTAGGGCTTAGGGGG via the pDONR^TM^221 vector as described above. Plasmids were verified by sequencing.

The negative control vector containing the minimal promoter only (*Hsp68/Venus*) was generated by annealing complementary oligos containing adjacent attB sequences (attbF1 GGGGACAAGTTTGTACAAAAAAGCAGGCTACCCAGCTTTCTTGTACAAAGTGGTCCCC, attbR1 GGGGACCACTTTGTACAAGAAAGCTGGGTAGCCTGCTTTTTTGTACAAACTTGTCCCC). These were made up to 800 ng/ul each in 1× TE and 50 mM NaCl and heated to 95°C before gradually cooling to room temperature. The double stranded attB sequence was then used in the recombination reactions described above in order to generate a destination vector containing only the attB sites upstream of the Hsp68/Venus reporter. All plasmid vectors generated as part of this study are available on request.

### ES cell culture

HM-1 cells (Magin et al., 1992) were cultured feeder-free on gelatinised tissue culture plates at 37°C in 5% CO_2_ in ES cell medium (Knockout Dulbecco's Modified Eagle Medium (KO-DMEM; Invitrogen) supplemented with 15% foetal bovine serum (Gibco), recombinant murine LIF (ORF Genetics), L-glutamine, Pen/Strep and β-mercaptoethanol).

### Gene targeting

Prior to gene targeting, HM-1 cells were cultured in ES cell medium supplemented with 6-thiguanine (Sigma) for 7 days to select for *Hprt*-deficient cells. HM-1 cells and linearised targeting vector were electroporated using a Gene Pulser electroporator (BioRad) at 800 V, 3 µF, and plated at clonal density. After 24 h, medium was replaced with ES cell medium supplemented with HAT (100 µM hypoxanthine, 0.4 µM aminopterin, 16 µM thymidine; Sigma) to select for *Hrpt* expressing clones. After 10–12 days, individual HAT-resistant ES cell colonies were picked and expanded in ES cell medium supplemented with HT (100 µM hypoxanthine, 16 µM thymidine; Sigma). ES cells were expanded in HT ES cell medium for seven days and then normal ES cell medium. Correct gene targeting was confirmed by Southern blotting or RT-PCR for expression of the reactivated *Hprt* using human_*HPRT*_exon1_forward CAGGCGAACCTCTCGGCTTT and mouse_*Hprt*_exon3_reverse GTGATGGCCTCCCATCTCCTT primers.

### Cardiac differentiation

Cardiac differentiation of ES cells was performed as described (Maltsev et al., 1993). Briefly ES cells were dissociated, washed twice and resuspended at a concentration of 400 cells/20 µl in Dulbecco's Modified Eagle Medium (Sigma) supplemented with 20% FCS (Hyclone), 5×10^−5^ M β-mercaptoethanol (Invitrogen) and 1× non-essential amino acids (Invitrogen). Cardiac differentiation by hanging drop method was initiated by pipetting 20 µl drops containing ES cells onto the lid of a 10 cm petri dish, which was then inverted and placed back on the dish containing PBS. Embryoid bodies were allowed to form in hanging drops for 2 days after which they were resuspended in 10 ml of culture medium in 10 cm petri dishes for a further 5 days. At day 7 of culture, single EBs were plated into individual wells of a 24 well plate and cultured for an additional 7 days. At day 14 of culture, individual EBs were scored for the presence of spontaneously contracting cells and expression of YFP.

### Haematopoietic differentiation

ES cells were differentiated into EBs according to (Sroczynska et al., 2009). Briefly, ES cells were passaged 24 h prior to differentiation and cultured in Iscove's Modified Dulbecco's Medium (IMDM; HyClone) supplemented with FBS, LIF and monothioglycerol (MTG). For the differentiation, ES cells were dissociated, washed twice and resuspended in differentiation medium (IMDM supplemented with 15% FBS (HyClone), 10% protein-free hybridoma medium II, 2 mM L-glutamine, 0.3 mg/ml human transferrin, 0.3 mM acorbic acid and 0.3 mM MTG). ES cells were seeded in Ultra Low Attachment 6-well dishes (Corning) at 10,000 cells/ml in 3 ml of differentiation medium. Differentiation medium was replaced after 5 days. EBs were harvested at required time points, disaggregated using Trp-LE (Invitrogen), spun down and resuspended in PBS supplemented with 5% FBS.

### Fluorescence imaging

Bright field and fluorescence images were taken using a LEICA DMI 3000B microscope with a Hamamatsu digital camera (Orca-Flash 4.0) and Optomorph (for still images) or HCLimageLive (for timelapse imaging) software.

### Flow cytometric analysis

Disaggregated EB cells were blocked using purified CD16/CD32 antibody (BD) and stained with PE-Cy7-CD41 antibody (Biolegend), APC-Flk1 antibody (BD), PE-CD140a (BD) or PE-CD41 (BD). Dapi (Sigma) was used as a viability stain. Cells were analysed using a 5 laser LSRFortessa cell analyser (BD) and data analysed using FlowJo software.

### Transgenic mouse breeding and embryo analysis

All mice were housed and maintained according to UK Home Office regulations. *Gata2^+/−^* mice (Tsai et al., 1994) and *Scl-3′enh/SV/lacZ* mice (Sánchez et al., 1999; Sánchez et al., 2001) were crossed and *Gata2^+/−^ Scl-3′enh/SV/lacZ* mice confirmed by genotyping. *Gata2^+/−^* and *Gata2^+/−^ Scl+19/SV/lacZ* mice were then crossed and E9.5 embryos collected, genotyped and stained as described previously (Tsai et al., 1994; Sánchez et al., 1999). The *Scl-3′enh* is a 5.5 kb genomic fragment that containing the 641 bp *Scl+19* enhancer element (Sánchez et al., 1999; Göttgens et al., 2002). The *Scl+19* element has previously been shown to be responsible for the activity of the *Scl-3′enh* region (Göttgens et al., 2002).

## Supplementary Material

Supplementary Material
