# Supplementary material for: Single site-specific integration targeting coupled with embryonic stem cell differentiation provides a high-throughput alternative to in vivo enhancer analyses
Source: Biol Open. 2013 Oct 7;2(11):1229–38. doi: 10.1242/bio.20136296 (PMC3828770; doi:10.1242/bio.20136296)
Supplement: Supplementary Material [file supp_2_11_1229__index.html]

Single site-specific integration targeting coupled with embryonic stem cell differentiation provides a high-throughput alternative to in vivo enhancer analyses — Supplementary Material 

# Single site-specific integration targeting coupled with embryonic stem cell differentiation provides a high-throughput alternative to in vivo enhancer analyses

## bio.20136296 Supplementary Material

**Files in this Data Supplement:**

- Supplementary Material - Adam C. Wilkinson et al. doi: 10.1242/bio.20136296
- Movie 1 - **Movie 1. Bright field (left) and fluorescent (right) time-lapse imaging of a representative HM-1 spontaneously beating day 14 EB.**
- Movie 2 - **Movie 2. Bright field (left) and fluorescent (right) time-lapse imaging of a representative *Hsp68/Venus* spontaneously beating day 14 EB.**
- Movie 3 - **Movie 3. Bright field (left) and fluorescent (right) time-lapse imaging of a representative *mm75/Hsp68/Venus* spontaneously beating day 14 EB.**
- Movie 4 - **Movie 4. Bright field (left) and fluorescent (right) time-lapse imaging of a representative *mm77/Hsp68/Venus* spontaneously beating day 14 EB.**
