# Supplementary material for: Single site-specific integration targeting coupled with embryonic stem cell differentiation provides a high-throughput alternative to in vivo enhancer analyses
Source: Biol Open. 2013 Oct 7;2(11):1229–38. doi: 10.1242/bio.20136296 (PMC3828770; doi:10.1242/bio.20136296)
Supplement: Supplementary Material [file supp_bio.20136296_bio.20136296-s1.pdf]

Supplementary Material

Adam C. Wilkinson et al. doi: 10.1242/bio.20136296

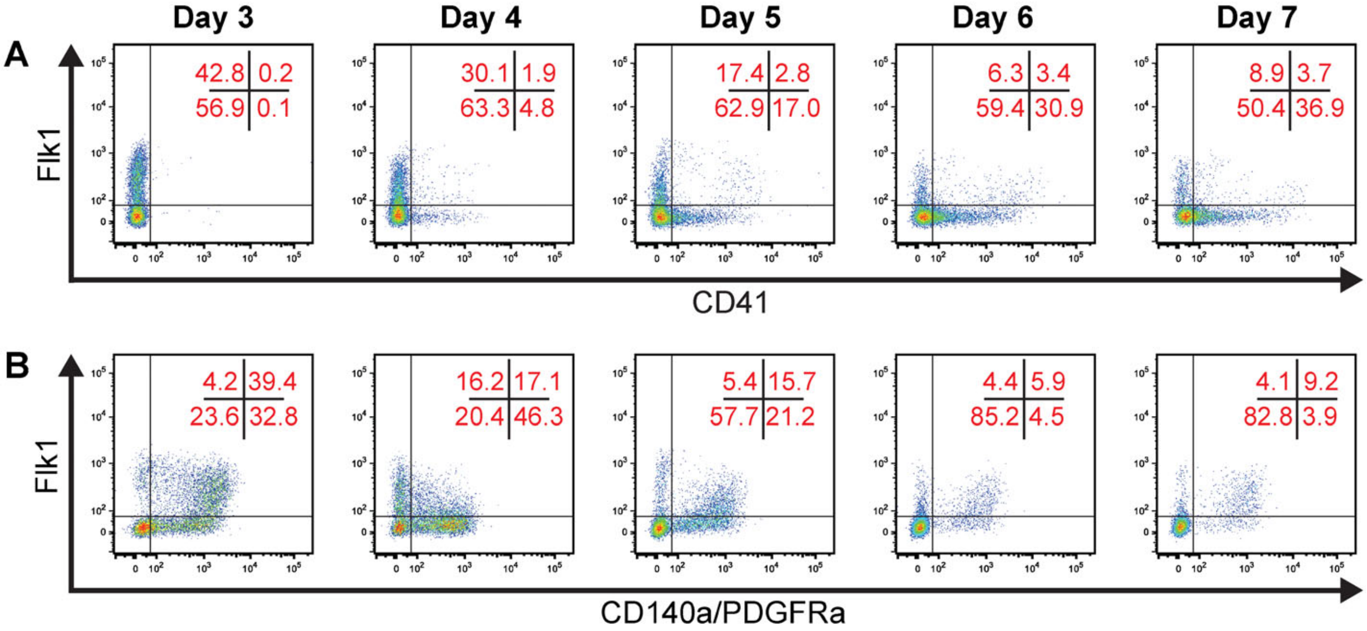

Fig. S1. Differentiation kinetics of HM-1 embryoid bodies. (A) Representative flow cytometry plots showing distribution of HM-1 EB cells in Flk1/CD41 quadrants from day 3–7. (B) As in (A) but for Flk1/CD140a quadrants.

Biology Open

Biology Open

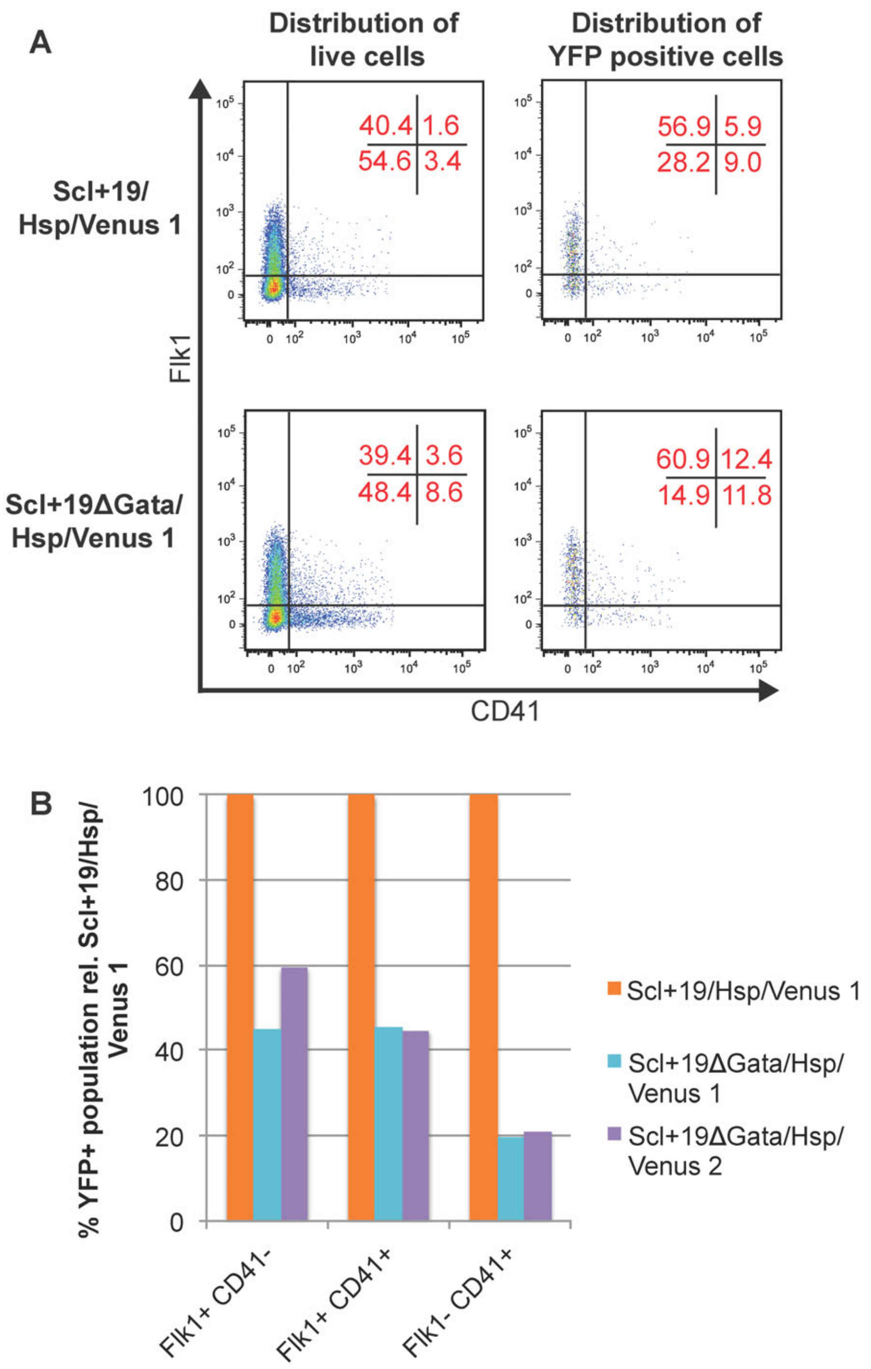

Fig. S2. Mutation of the Gata motif in the *Scl+19* enhancer results in loss of YFP+ cells in all Flk1/CD41 quadrants in day 4 EBs. (A) Representative flow cytometry plots showing distribution of *Scl+19/Hsp/Venus* (above) *Scl+19ΔGata/Hsp/Venus* (below) day 4 EB cells in Flk1/CD41 quadrants for all live cells (left plot) and YFP positive cells only (right plot), with the percentage of cells in each quadrant shown in red. (B) Percentage of YFP positive cells in Flk1/CD41 quadrants for *Scl+19ΔGata/Hsp/Venus* clones (clone 1 in blue, clone 2 in purple) relative to *Scl+19/Hsp/Venus* clone 1 (in red). Average of two independent differentiation experiments.

Table S1. ES cell differentiation potential using embryoid bodies. A summary of germ layers, precursors and progenitors, and mature cell types that can be produced by embryoid body differentiation as outlined in Höpfl et al., 2004.

Germ layer	Precursor and progenitors	Mature cells
Mesoderm	Haematopoietic precursor and progenitor	Primitive erythroid cells<br>Definitive erythoid cells<br>Mast cell<br>Macrophage<br>Dendritic cell<br>Megakaryocyte<br>Lymphoid cell
		Endothelium
	Cardiac precursor and progenitor	Cardiomyocytes
	Muscle progenitor	Smooth muscle
		Chrondocytes<br>Osteoblast
Endoderm		Pancreatic cell<br>Hepatocyte
Ectoderm		Adipocyte<br>Neuron

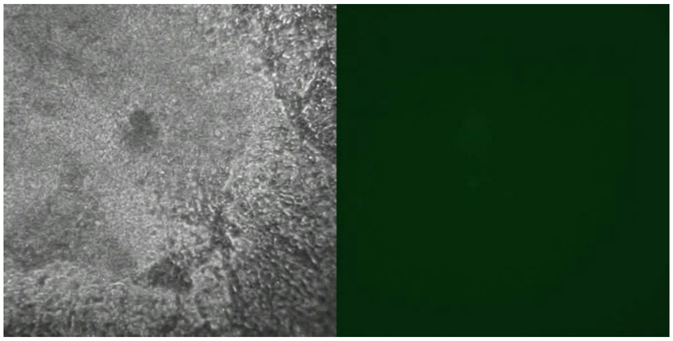

Movie 1. Bright field (left) and fluorescent (right) time-lapse imaging of a representative HM-1 spontaneously beating day 14 EB.

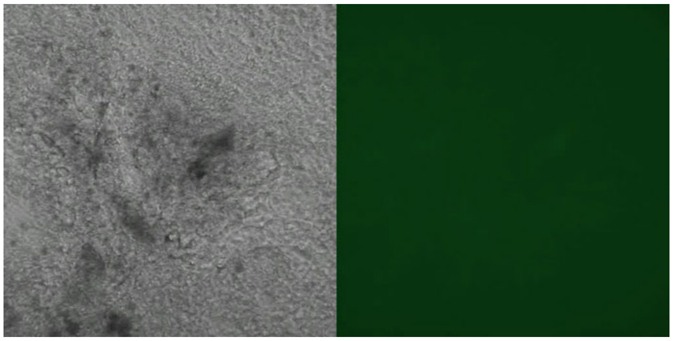

Movie 2. Bright field (left) and fluorescent (right) time-lapse imaging of a representative *Hsp68/Venus* spontaneously beating day 14 EB.

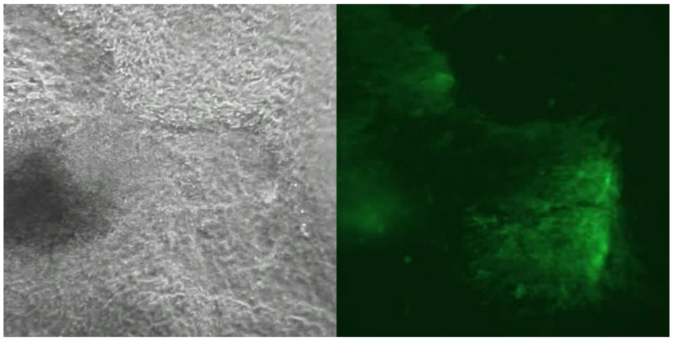

Movie 3. Bright field (left) and fluorescent (right) time-lapse imaging of a representative *mm75/Hsp68/Venus* spontaneously beating day 14 EB.

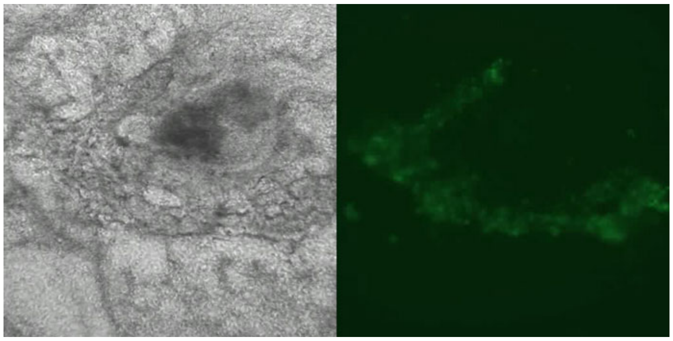

Movie 4. Bright field (left) and fluorescent (right) time-lapse imaging of a representative *mm77/Hsp68/Venus* spontaneously beating day 14 EB.
